# Enhanced Frequency Stability of SAW Yarn Tension Sensor by Using the Dual Differential Channel Surface Acoustic Wave Oscillator

**DOI:** 10.3390/s23010464

**Published:** 2023-01-01

**Authors:** Yang Feng, Wenbo Liu, Ben Wang

**Affiliations:** School of Information Science and Technology, Hangzhou Normal University, Hangzhou 311121, China

**Keywords:** surface acoustic wave (SAW), yarn tension sensor, dual differential channel circuit, oscillators frequency stability

## Abstract

This paper presents a 60 MHz surface acoustic wave (SAW) yarn tension sensor incorporating a novel SAW oscillator with high-frequency stability. A SAW delay line was fabricated on ST-X quartz substrate using the unbalanced-split electrode and bi-directional engraving slots. The dual differential channel delay linear acoustic surface wave oscillator is designed and implemented to test yarn tension, which can effectively remove the interference of temperature, humidity, and other peripheral factors through differential design. The yarn tension sensor using the surface acoustic wave has high-precision characteristics, and the SAW delay line oscillator is designed to ensure the test system’s stable operation. The effect of time and tension on oscillator frequency stability is studied in detail, and the single oscillator and the dual differential channel system were tested, respectively. After using the dual differential channel system, the short-term frequency stability from is reduced from 1.0163 ppm to 0.17726 ppm, the frequency accuracy of the tension sensor is improved from 134 Hz to 27 Hz, and the max frequency jump steady is reduced from 2.2395 ppm to 0.45123 ppm.

## 1. Introduction

The yarn tension is an essential indicator of measuring the quality of the yarn product, which directly affects the balance and stability of the product quality, the production efficiency, and the subsequent processing [1]. Appropriate yarn tension makes the winding bobbin roll efficient, increasing the production efficiency of winding, twisting, weaving, and knitting [2,3,4]. Therefore, selecting an appropriate detection circuit is necessary to ensure the precision and stability of the yarn tension sensor’s output signal [5].

At present, the commonly used sensors for detecting yarn tension are based on phase detection systems [6], amplitude detection systems [7], the phase lock loop (PLL) detection method [8], direct digital synthesizer (DDS) scanning detection [9], and mixing detection method [10]. Among them, phase detection and amplitude detection systems are used in the largest amounts; however, both of them are low sensitivity [11]. The way to select the PPL and DDS circuit makes the detection circuits very complicated [12]. In addition, to simplify the detection circuits and obtain high precision and stable detection signals, the mixing detection circuit is presented [13].

The yarn tension sensor is based on the surface acoustic wave device and oscillation circuit. Two factors affect the frequency stability of the SAW oscillator (SAWO): one is the performance of the SAW device, and the other is the oscillation circuit’s noise [14,15,16]. This paper focused on the frequency stability of SAWO and solved the following two critical problems:

(1) The following measures are taken to minimize the second order of the surface acoustic wave devices. First, this is accomplished by reducing the electrode reflection by using the unbalanced-split electrode [17]. Second, this is accomplished by reducing the sound–electricity reclamation (SER) by choosing the ST-X quartz as the piezoelectric substrate [18]. Third, reducing the interference of bulk acoustic wave (BAW) by engraving bi-directional engraving slots of the piezoelectric substrate [19].
(2) To solve the temperature interference, the dual differential channel SAWO is designed. The dual differential channel SAWO consists of the double delay line oscillators, an integrated mixer circuit, and the LC low pass filter. At the same time, the signals source circuit and voltage regulator circuits are designed, and then Agilent E5061A ENA-L was used to test and analyze the PCB board.

This paper is organized as follows. After this introductory Section 1, Section 2 presented the principle of the SAWO dual differential channel circuit. The design and preparation of the SAW yarn tensor sensor are shown in Section 3. In Section 4, the test and application of the dual differential channel of the SAWO are given. Conclusions are drawn in Section 5.

## 2. Principle of the SAWO Dual Differential Channel Circuit

**Figure 1:**
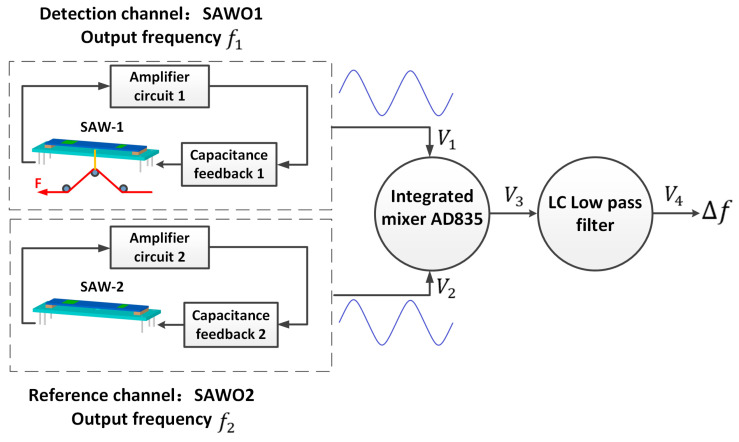
The schematic of SAW yarn tension sensor with dual differential circuit system.

Figure 1 shows the dual differential surface acoustic wave oscillator system. It can be divided into three parts:

(1) Two identical SAWOs. One of the SAWOs applies yarn tension on the piezoelectric substrate, namely, the detection channel; another SAWO is the reference channel.
(2) An integrated mixer. When the output frequency signals of the two SAWO are added to the mixer, the output signal of the mixer is the frequency sum and frequency difference.
(3) LC low-pass filter. When the output signal of the mixer passes through the low-pass filter, the output signal of the low-pass filter is the frequency difference (that is, the frequency difference in the output signal of the mixer).

In Figure 1, SAWO1 generate the sine wave signal *V*_1_, and the output frequency is $f_{1}$. SAWO2 create the sine wave signal *V*_2_, and the output frequency is $f_{2}$. Signals *V*_1_ and *V*_2_ entered into the integrated mixer AD835, and the signal output frequency is *V*_3_, where *V*_3_ is the frequency sum and frequency difference of *V*_1_ and *V*_2_ ($V_{1}\pm V_{2}$). After the mixed output, signal *V*_3_ is input to the LC low pass filter, the frequency sum of $f_{1}$ and $f_{2}$ are filtered out, and only the frequency difference of $f_{1}$ and $f_{2}$ is left.

In this system, SAW1 is used to measure the yarn tension, and SAW2 is used as a reference. The output signals $V_{1}$ of the SAWO1 can be expressed as:

(1) $$V_{1}=\sqrt{2}U_{1}\sin(2\pi f_{1}t+\varphi_{1})$$

where $U_{1}$ is the effective value of the output voltage of SAWO1, $f_{1}$ is the frequency of the output signal of the SAWO1, and $\varphi_{1}$ is the initial phase angle of the output signal of SAWO1.

The output signal $V_{2}$ of the SAWO2 can be expressed as:

(2) $$V_{2}=\sqrt{2}U_{2}\sin(2\pi f_{2}t+\varphi_{2})$$

where $U_{2}$ is the effective value of the output voltage of SAWO2, $f_{2}$ is the frequency of the output signal of the SAWO2, and $\varphi_{2}$ is the initial phase angle of the output signal of SAWO2.

The mixing circuit adopts an integrated mixer AD835 as the core signal processing circuit. AD835 is a voltage output multiplier produced by analog devices, which can complete the function of W=XY+Z. The X and Y input signals range from −1 V to +1 V, and the bandwidth is up to 250 MHz. It is suitable for mixing the output signals of dual SAWO.

When the output signals $V_{1}$ and $V_{2}$ pass through the integrated mixer AD835 circuit, the signals of the two SAWOs will be mixed, and the signal $V_{3}$ after mixing is:

(3) $$\begin{array}{l} V_{3}=V_{1}\times V_{2}\\ \;\;\;\;=\left[\sqrt{2}U_{1}\sin(2\pi f_{1}t+\varphi_{1})\right]\left[\sqrt{2}U_{2}\sin(2\pi f_{2}t+\varphi_{2})\right]\\ \;\;\;\;=2U_{1}U_{2}\left\{\cos\left[2\pi (f_{1}-f_{2})+(\varphi_{1}-\varphi_{2})\right]-\cos\left[2\pi (f_{1}+f_{2})+(\varphi_{1}+\varphi_{2})\right]\right\} \end{array}$$

In Equation (3), the mixing signal contains $f_{1}-f_{2\;}$ and $f_{1\;}+f_{2\;}$ signals. According to Figure 1, the mixer output signal $V_{3}$ passes through the low-pass filter, and the output signal $V_{4}$ is:

(4) $$V_{4}=K_{L}U_{1}U_{2}\cos\left[2\pi (f_{1}-f_{2})t+(\varphi_{1}-\varphi_{2})\right]$$

where $K_{L}$ is the magnification of the low pass filter.

The simultaneity of the input supply voltage *Vi* applied to the dual surface acoustic wave oscillator system and the symmetry of the system, concerning the initial phase of the SAWO1 and the SAWO2, should be:

(5) $$\varphi_{1}=\varphi_{2}$$

By substituting Equation (5) into Equation (4), the output signal $\;V_{4}$ is converted into:

(6) $$V_{4}=K_{L}U_{1}U_{2}\cos\left[2\pi (f_{1}-f_{2})t\right]==KU_{1}U_{2}\cos\left(2\pi \Delta f_{LPF}t\right)$$

where $\Delta f_{LPF}$ is the frequency difference of SAWO1 and SAWO2.

According to Equation (6), when the dual differential SAWO is affected by external interference, the output signal is still $\Delta f_{LPF}=f_{1}-f_{2}$. So, the design of a dual channel can suppress external interference to a certain extent and realize the stability and anti-interference of the system.

However, in practice, because the device structure cannot be completely symmetric, the component distribution is somewhat different, and the device parameters cannot be entirely consistent, so the difference between the frequency $f_{1}$ of the SAWO1 and the $f_{2}$ of the SAWO2 will not be utterly equal to zero. Therefore, before the test, it is necessary to determine the essential calibration value $f_{00}\;$ of the detection and the reference channels.

## 3. Design and Preparation of the SAW Yarn Tensor Sensor

### 3.1. SAW Delay Line

As the core of SAWO, the frequency response of SAW will directly affect the performance of the whole circuit. For the excellent frequency characteristics of SAW devices, it is necessary to select a piezoelectric substrate with a high electromechanical coupling coefficient and improve the side lobe and bulk acoustic wave suppression ability. Therefore, the SAW devices are optimized from the following three aspects.

**Figure 2:**
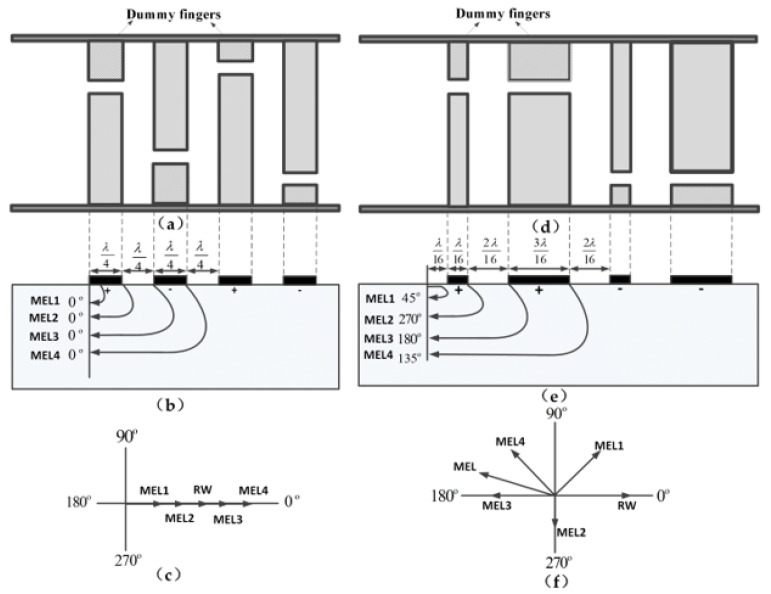
Electrode reflection of single electrodes IDTs and unbalanced-split-electrode IDTs. (**a**) Single electrodes IDTs; (**b**) Mass load (MEL) reflection of single electrodes IDTs; (**c**) Phase synthesis diagram of single electrodes IDTs; (**d**) Unbalanced-split electrodes IDTs; (**e**) Mass load (MEL) reflection of unbalanced-split electrodes IDTs; (**f**) Phase synthesis diagram of unbalanced-split electrodes IDTs.

(1) Choosing the unbalanced-split electrode to solve the electrode reflection and side lobe of the SAW devices, as shown in Figure 2.

**Figure 3:**
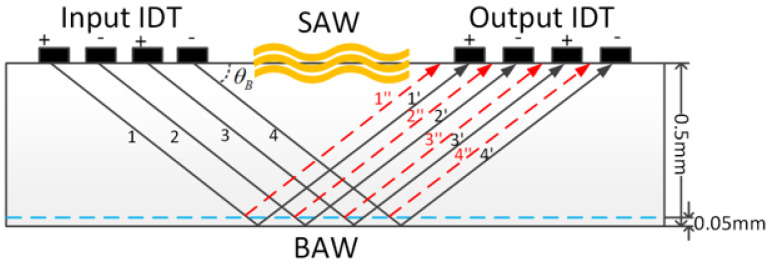
Change in BAW reflection excited by IDT by engraving bi−directional slots.

Single electrode IDT is arranged in a periodic λ/2 (Figure 2a), and the regenerated waves (RW) are caused by the metal electrode. In Figure 2b, MEL1, MEL2, MEL3, and MEL4 are the mass/electrical load reflection reflected by the edge of each metal electrode, which is of the same phase. Therefore, the electrode reflection received by the centre of the transducer is the sum of all electrode reflections, as shown in Figure 3c. The unbalanced-split electrode width is λ/16 and 3λ/16, with an interval of 2λ/16, as shown in Figure 2d. Figure 2e is the phase synthesis diagram of the unbalanced-split-electrode interdigital transducers (IDT). The total phase of the regenerated reflection wave and mass load feedback is close to 180° (Figure 2f), effectively reducing the in-band ripple effect characterised by the sensor frequency response.

(2) Choosing the ST-X Quartz as the piezoelectric substrate to decrease SER.

The electromechanical coupling coefficient $k^{2}$ determines the suitable material for the SAW sensor. $k^{2}$ represents the energy conversion degree of SAW piezoelectric material, which is related to SER [20]. The larger the $k^{2}\;$ is, the stronger the SER is. Therefore, the minor $k^{2}\;$ material is chosen to ensure the IDTs of the SAW sensor can perform well. The common materials of the piezoelectric substrate and their $k^{2}$ values are listed in Table 1.

(3) Engraving bi-directional slots on the back of the piezoelectric substrate to solve the interference of BAW in SAW devices, as shown in Figure 3.

The engraved bi-directional slots on the back of the substrate can block the propagation path of BAW to a certain extent, reducing the influence of BAW propagation and suppressing the out-of-band suppression of the frequency response. In Figure 3, the way of 1 is the electrode-A’s BAW, the way of 1’ is the reflection of DBAW before the slotting, and the way of 1’ (red dotted line) is the reflection of DBAW after the slotting. After slotting, the thickness of the piezoelectric substrate changes (from 0.5 mm to 0.45 mm, slot depth of 0.05 mm), which leads to the most potent reflection of DBAW falls on the end of the central area output IDT, thus significantly weakening the influence of DBAW.

**Figure 4:**
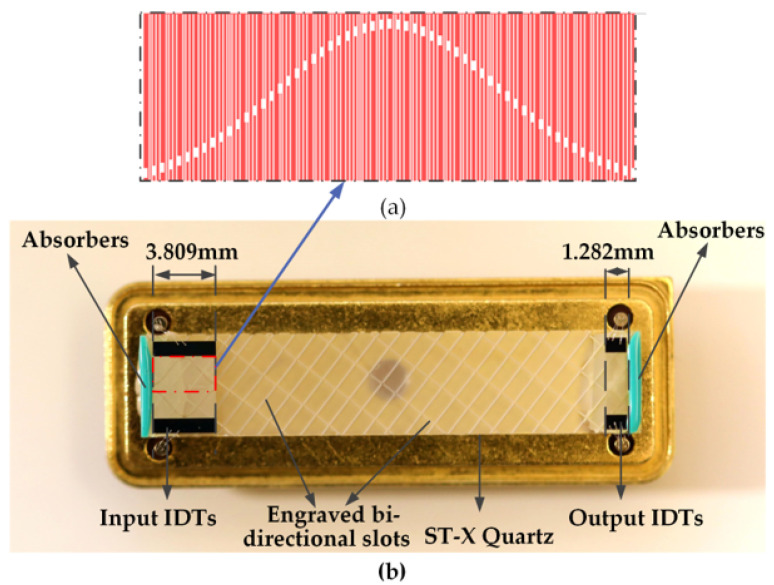
Surface acoustic wave devices fabricated on ST-X Quartz substrates. (**a**) unbalanced-split electrode (**b**) image of SAW devices.

The SAW design parameters are shown in Table 2 and fabricated on ST-X quartz substrates, as shown in Figure 4.

### 3.2. Dual Differential Channel Circuit

There are two kinds of structures of the surface acoustic wave oscillator constructed by transistor: Pierce type and Colpitts type low noise oscillation circuit [21]. The crystal and the inductor of the Pierce oscillator are connected in series to form a series resonant circuit, which works on the series resonance [22]. The Colpitts oscillator is a parallel resonant circuit with large total impedance and low-frequency stability [23]. This paper used the surface acoustic wave device as the oscillator frequency output device, and the working frequency is high. Therefore, choosing the Pierce oscillator can improve the frequency stability of the surface acoustic wave device.

**Figure 5:**
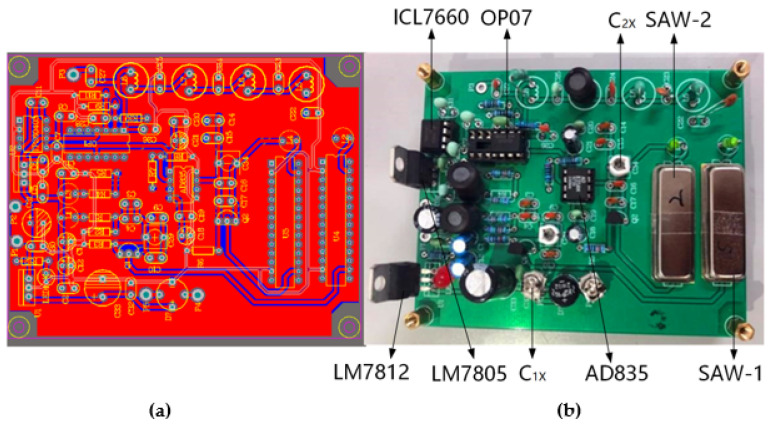
Diagram of the dual differential surface acoustic wave oscillator system. (**a**) The design of PCB. (**b**) The Circuit board.

The dual differential surface acoustic wave oscillator system is shown in Figure 5. The power supply voltage of the oscillating circuit is +12 V, and the positive and negative power supply voltages of the mixing module AD835 are, respectively, ±5 V. +5 V is generated by +12 V voltage through LM7805. Figure 5a shows the PCB layout of the circuit of the yarn tension sensor. The PCB size is 100 mm × 100 mm. In Figure 5b, C_1X_ and C_2X_ is the adjustable capacitor whose purpose is to determine the basic calibration value of the detection and the reference channels. AD835 is a mixer of ADI Semiconductor company. Its operating frequency is 250 MHz, which meets the frequency requirement of 60 MHz of SAW device design.

## 4. Test and Application of the Dual Differential Channel of the SAWO

### 4.1. Test of SAW Delay Line

**Figure 6:**
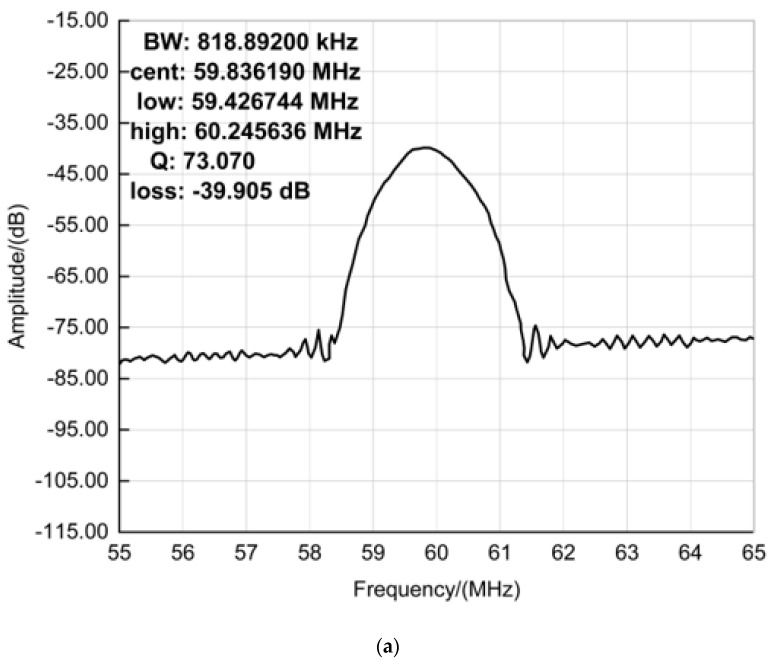

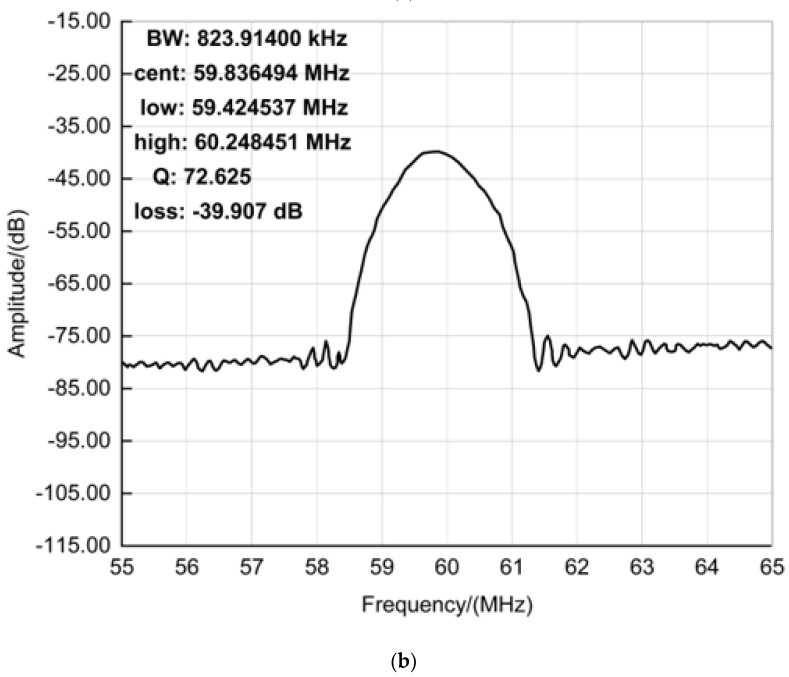
Frequency characteristics of the acoustic surface wave devices. (**a**) SAW−1; (**b**) SAW−2.

Agilent E5061A ENA-L radio frequency network analyzer was used to test the acoustic surface wave devices’ frequency response characteristics. The frequency response of a group of acoustic surface wave devices was measured, as shown in Figure 6. Figure 6a shows the frequency characteristics of SAW-1, whose centre frequency is 59.836190 MHz. Figure 6b shows the frequency characteristics of SAW-2, whose centre frequency is 59.836494 MHz.

### 4.2. Test and Analysis of Dual Differential Channel Circuit

#### 4.2.1. The Basic Calibration Value of the Detection Channel and Reference Channel

**Figure 7:**
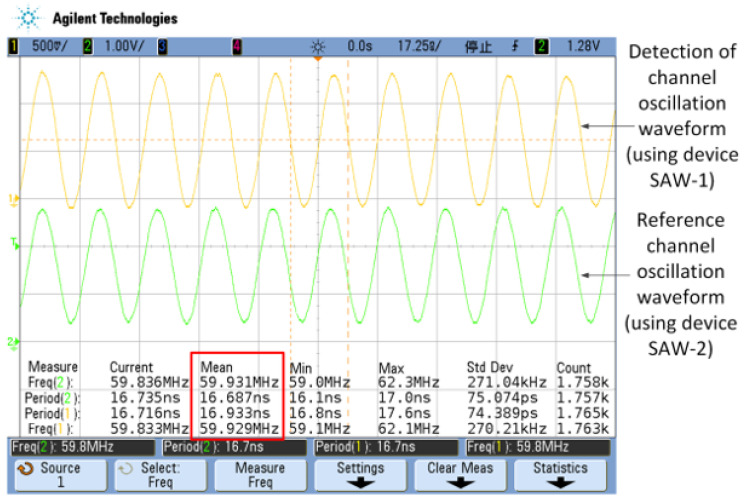
Surface acoustic wave oscillator waveform diagram (Test by KEYSIGHT 4000X).

KEYSIGHT 4000X high-performance hybrid digital oscilloscope (4 channels 200 MHz) is used to test the frequency of SAW devices, and the oscillation waveform is shown in Figure 7. In Figure 7, the yellow curve is the detection channel’s waveform, and the green curve is the reference channel’s waveform. Since the circuit parameters of the two channels are the same, the oscillation frequency of both channels is 59.8 MHz (shown in the red box of Figure 7).

When the environment temperature changes, the frequency of the output signal of surface SAWO2 is:

(7) $$f_{2}=f_{02}+\Delta f_{T2}$$

where $f_{02}\;$ is the centre frequency of SAWO2; $\Delta f_{T2}$ is the frequency variation of surface SAWO2 caused by temperature change.

At the same time, the frequency of the output signal of SAWO1 is:

(8) $$f_{1}=f_{01}+\Delta f_{T1}+\Delta f_{F}$$

where $f_{01}\;$ is the centre frequency of SAWO1; $\Delta f_{T1}$ is the frequency variation of SAWO1 caused by temperature change. $\Delta f_{F}$ is the centre frequency change in SAWO1 when yarn tension F≠0.

The output frequency of the low-pass filter is:

(9) $$\Delta f_{LPF}=f_{1}-f_{2}$$

By substituting Equations (7) and (8) into Equation (9), one obtains:

(10) $$\begin{array}{l} \Delta f_{LPF}=\left(f_{01}+\Delta f_{T1}+\Delta f_{F}\right)-\left(f_{02}+\Delta f_{T2}\right)\\ \;\;\;\;=\left(f_{01}-f_{02}\right)+\left(\Delta f_{T1}-\Delta f_{T2}\right)+\Delta f_{F}\\ \;\;\;\;=f_{00}+\Delta f_{T}+\Delta f_{F} \end{array}$$

where $f_{00\;}$ is a constant, $f_{00}=f_{01}-f_{02}$, and $\Delta f_{T}$ is the frequency difference between the two oscillators caused by temperature.

To determine $f_{00}$ in Equation (10), the basic calibration values of the detection channel and the reference channel should be tested. The determination method is given below.

**Figure 8:**
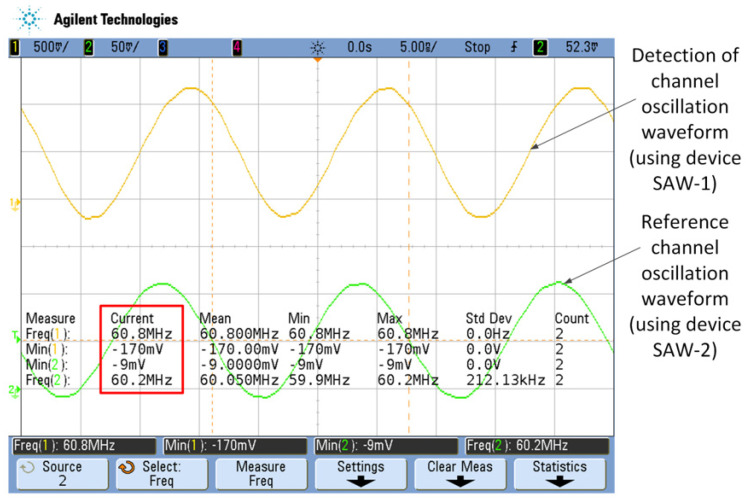
Test waveform diagram of the surface acoustic wave oscillator after adjusting the capacitor Cx.

Adjust the capacitor Cx (in Figure 5b) to offset the oscillation frequency of the detection channel and the reference channel. This offset is a fixed value and does not change with environmental conditions. In this experiment, this offset value is defined as the basic calibration value η of the frequency difference output, which is the difference between the oscillation frequencies of the yellow curve and the green curve in Figure 8 (shown in the red box).

When the yarn tension F is loaded into the detection channel, the difference frequency output of the yarn tension sensor circuit is subtracted from the basic calibration value *η*. That is, the frequency difference component $\Delta f_{LPF}$ is obtained.

#### 4.2.2. Output Signal of Low Pass Filter

**Figure 9:**
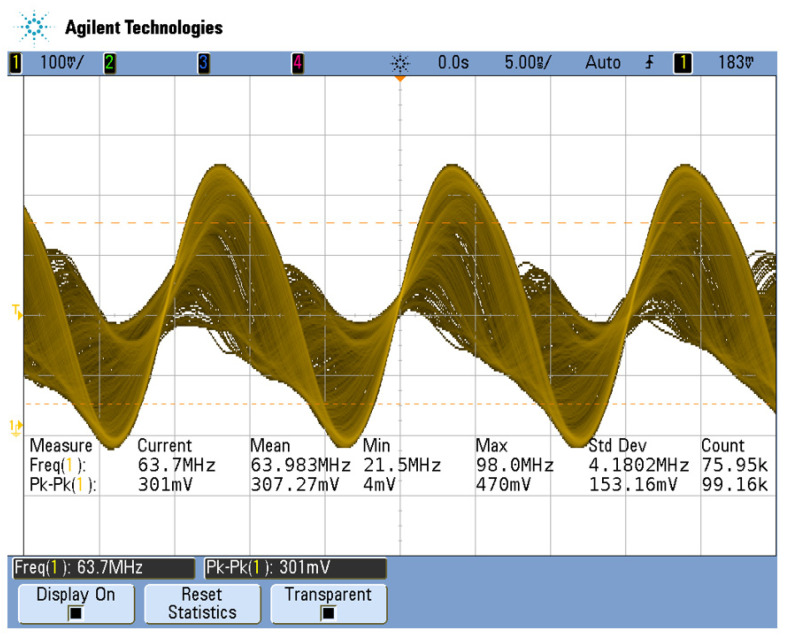
Test waveform diagram of the output signal of AD835 mixer.

The mixing waveform is measured after the mixer AD835, as shown in Figure 9. Due to the complex harmonic components, disorderly waveforms, and broad-spectrum range in the waveform shown in Figure 9, differential frequency components with lower frequencies need to be extracted.

**Figure 10:**
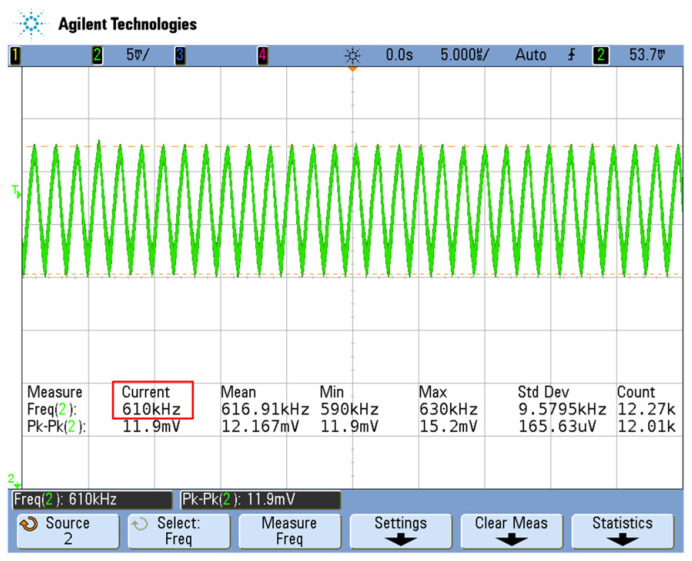
Test waveform diagram of differential frequency signal of low-pass filter.

Figure 10 shows the output waveform after the second-order low-pass filter is passed. The difference frequency signal is a sine wave signal, and the oscillation frequency is 610 kHz (as shown in the red box in Figure 10), which is the difference frequency value of the two waveforms in Figure 8 (as shown in the red box). Therefore, the basic calibration value of the measured detection channel and reference channel η=610 kHz. That is, $f_{00}=610\;\mathrm{kHz}$, and Equation (10) can be changed to:

(11) $$\Delta f_{LPF}=610k+\Delta f_{T}+\Delta f_{F}$$

In summary, after the complex harmonic signal output by the mixer passes through the low-pass filter, the high-frequency harmonic in the signal is significantly attenuated, and only a single component of the difference frequency signal is left. Thus, the detection function of the difference circuit is realized, and the yarn tension sensor can eliminate the influence of environmental interference.

### 4.3. Test and Analysis of Dual Differential Channel Circuit Stability

The stability of the oscillating circuit is an essential factor in determining the performance of a surface acoustic wave sensor. The frequency stability of SAWO refers to the random frequency variation value within a specific sampling time, which can be divided into long-term, medium-term, and short-term frequency stability. There are two ways to express short-term frequency stability: one is the time domain representation, which is generally expressed by Allen variance; the other is frequency domain representation, which can be represented by phase noise. The Allen variance is commonly used to describe short-term frequency stability, which is defined as:

(12) $$\sigma (\tau )=\sqrt{\frac{1}{2N}\sum_{k=1}^{N}{\left(f_{k+1}-f_{k}\right)}^{2}}$$

where τ  is the sampling interval, $f_{k}\;$ is the frequency point, and *N* is the total of samples.

In the experiment, the interval sampling is *k* times, and short-term frequency stability can be defined as:

(13) $$K=\frac{\delta (t)}{f_{M}}=\frac{1}{f_{M}}{\left[\frac{{\sum_{k=1}^{N}\left(f_{k+1}-f_{k}\right)}^{2}}{2N}\right]}^{\frac{1}{2}}$$

where $f_{M}$ is the average frequency. Equation (13) *K* is used to estimate the short-term frequency stability of the oscillating circuit.

#### 4.3.1. Frequency Stability of SAWO1 Output Signal with Loaded Tension

**Figure 11:**
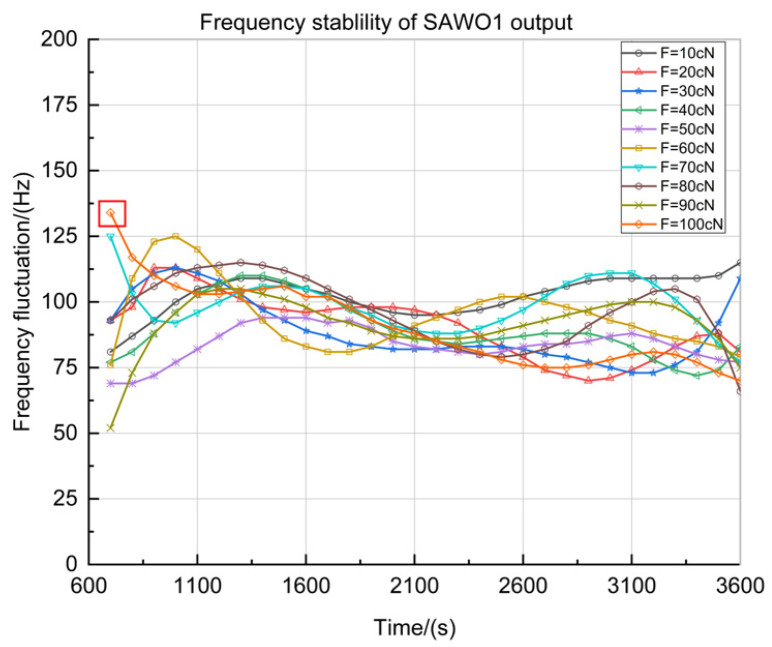
Frequency stability curve of SAWO1 loaded with tension (the red box is the maximum data for all the tests).

The output of SAWO1 with different tension from 0 to 100 cN is tested at intervals of 10 cN. A continuous test was carried out for one hour, and the frequency fluctuation was recorded between 600 s and 3600 s. As shown in Figure 11, ten groups of data ranging from 10 cN to 100 cN are obtained from 600 s to 3600 s.

In Figure 11, the *X*-axis is the measuring period from 600 s to 3600 s, frequency sampling is conducted every 100 s, and the *Y*-axis is the value of the frequency fluctuation near the center frequency. According to the test data in Figure 11 and the centre frequency of SAW-1 ($f_{S1-M}=59.836190\;\mathrm{MHz}$ in Figure 6a), the short-term frequency stability can be obtained as:

(14) $$K_{S1}=\frac{1}{f_{S1-M}}{\left[\frac{{\sum_{k=1}^{N}\left(f_{k+1}-f_{k}\right)}^{2}}{2N}\right]}^{\frac{1}{2}}=1.0163\times 10^{-6}=1.0163ppm$$

Table 3 shows the data with the largest frequency fluctuation among the ten data groups, measured when F=100 cN tension. According to the test data in Table 3, the max frequency jump of the detection circuit can be stable at 134 Hz (time at 700 s) from 600 s to 3600 s (Figure 11 in red box), so the max frequency jump is steady at:

(15) $$K_{S1-\max}=\frac{\Delta f_{\delta \max}}{f_{S1-M}}=\frac{134\mathrm{Hz}/h}{59.83619\mathrm{MHz}}=2.2395\times 10^{-6}=2.2395\mathrm{ppm}$$

#### 4.3.2. Frequency Stability of Low Pass Filter Output Signal with Loaded Tension

**Figure 12:**
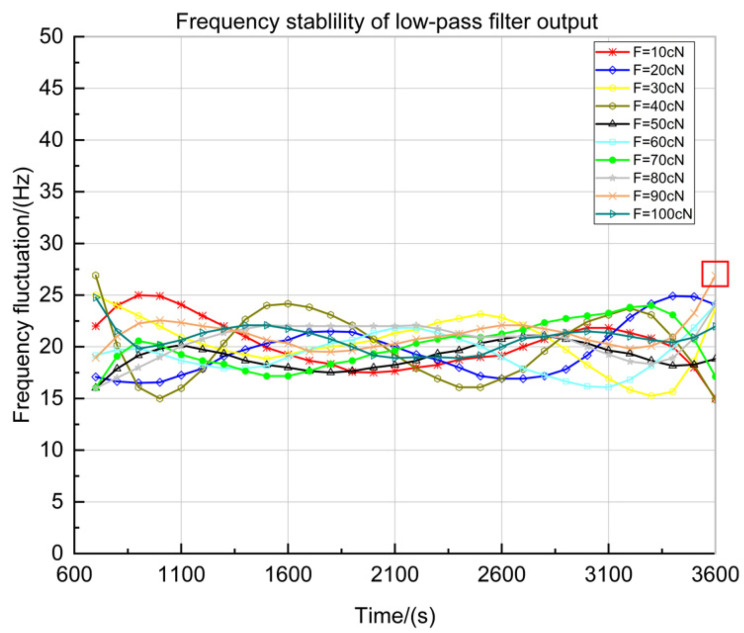
Frequency stability curve of low-pass filter output loaded with tension (the red box is the maximum data for all the tests).

The output of low pass filter with different tension from 0 to 100 cN also is tested at intervals of 10 cN. A continuous test was carried out for one hour, and the frequency fluctuation was recorded between 600 s and 3600 s. As shown in Figure 12, ten groups of data ranging from 10 cN to 100 cN are obtained from 600 s to 3600 s.

In Figure 12, the *X*-axis is the measuring period from 600 s to 3600 s, frequency sampling is conducted every 100 s, and the *Y*-axis is the value of the frequency fluctuation near the center frequency. According to the test data in Figure 12 and the centre frequency of SAW-2 ($f_{S2-M}=59.836494\;\mathrm{MHz}$ in Figure 6b), the short-term frequency stability can be obtained as:

(16) $$K_{S2}=\frac{1}{f_{S2-M}}{\left[\frac{{\sum_{k=1}^{N-1}\left(f_{k+1}-f_{k}\right)}^{2}}{2N}\right]}^{\frac{1}{2}}=1.7726\times 10^{-7}=0.17726\mathrm{ppm}$$

Table 4 shows the data with the largest frequency fluctuation among the ten data groups, measured when F=90 cN tension. According to the test data in Table 4, the max frequency jump of the detection circuit can be stable at 27 Hz (time at 3600 s) from 600 s to 3600 s (Figure 12 in red box), so the max frequency jump is steady at:

(17) $$K_{S2-\max}=\frac{\Delta f_{\delta \max}}{f_{S2-M}}=\frac{27\mathrm{Hz}/h}{59.836494\mathrm{MHz}}=4.5123\times 10^{-7}=0.45123\mathrm{ppm}$$

## 5. Conclusions

This paper presents a design for the dual differential channel SAWO to enhance the SAW yarn tension sensor’s frequency stability. The method of surface acoustic wave devices is optimized from three aspects. First, this involves designing the unbalanced-split electrode to reduce the electrode reflection and side lobe of the oscillator. Second, this involves choosing the ST-X Quartz as the piezoelectric substrate to reduce the SER. Third, this involves engraving the back grooving of the piezoelectric substrate to reduce the interference BAW.

The dual differential channel circuits are designed and manufactured by using an AD835 mixer. The output of SAWO1 and low pass filter with different tension from 0 to 100 cN are tested at intervals of 10 cN. A continuous test was carried out for one hour, and the frequency fluctuation was recorded between 600 s and 3600 s.

The conclusions were shown as follows:

(1) The dual differential channel SAWO can enhance the frequency stability of the SAW yarn tension sensor.
(2) Using the dual differential channel SAWO can reduce the short-term frequency stability from 1.0163 ppm to 0.17726 ppm.
(3) The frequency accuracy of the tension sensor is improved from 134 Hz to 27 Hz.
(4) The max frequency jump steady is reduced from 2.2395 ppm to 0.45123 ppm.

## Figures and Tables

**Table 1:** Piezoelectric substrate materials and their $k^{2}$.

Material	SiO_2_	SiO_2_	SiO_2_	LiTaO_3_	LiTaO_3_	LiTaO_3_
Direction of propagation	ST-X	42.75^0^Y-X	Y-X	Y-Z	X-112^0^Y	42^0^Y-X
$k^{2}/\%$	0.14	0.16	0.23	0.66	0.75	7.6
Material	LiNbO_3_	LiNbO_3_	LiNbO_3_	Li_2_B_4_O_7_	Li_2_B_4_O_7_	Li_2_B_4_O_7_
Direction of propagation	X-112^0^Y	Z-X	128^0^Y-X	Z-X	Z-45^0^X	6.7^0^X-Z
$k^{2}/\%$	0.75	2.0	5.5	0.9	1.1	1.4

**Table 2:** Design parameters of SAW delay line.

Piezoelectric Substrate	Material	ST-X Quartz
	Size	L = 30 mm, W = 6 mm, H = 0.5 mm
IDT	Structure	Delay line
	The centre frequency	60 MHz
	−3 dB bandwidth	1.38%
	Wavelength Aperture width	λ=52.633333 μm 3588.733333 μm
	Number of input IDT	145
	Number of output IDT	49
	Distance between input and output IDT center	27.422 mm(521 λ)

**Table 3:** Frequency fluctuations test data of SAWO1 from 600 s to 3600 s when *F* = 100 cN (the group where the greatest fluctuation occurred, and the red color is the maximum data for this group of tests).

Time(s)	600	700	800	900	1000	1100	1200	1300	1400	1500	1600
$\Delta f_{\delta }$(Hz)	0	134	117	110	106	103	103	104	105	106	102
Time(s)	1700	1800	1900	2000	2100	2200	2300	2400	2500	2600	2700
$\Delta f_{\delta }$(Hz)	102	98	93	90	88	85	83	81	78	76	75
Time(s)	2800	2900	3000	3100	3200	3300	3400	3500	3600		
$\Delta f_{\delta }$(Hz)	75	76	78	80	81	80	77	73	70		

**Table 4:** Frequency fluctuations test data of low-pass filter output from 600 s to 3600 s when F=90 cN (the group where the greatest fluctuation occurred, and the red color is the maximum data for this group of tests).

Time(s)	600	700	800	900	1000	1100	1200	1300	1400	1500	1600
$\Delta f_{\delta }$(Hz)	0	19	21	22	23	22	22	22	21	21	20
Time(s)	1700	1800	1900	2000	2100	2200	2300	2400	2500	2600	2700
$\Delta f_{\delta }$(Hz)	20	19	20	20	20	21	21	21	22	22	22
Time(s)	2800	2900	3000	3100	3200	3300	3400	3500	3600		
$\Delta f_{\delta }$(Hz)	22	21	21	20	20	20	21	23	27		

## Data Availability

Not applicable.
